# Wilson protein expression, copper excretion and sweat production in sweat glands of Wilson disease patients and controls

**DOI:** 10.1186/1471-230X-8-29

**Published:** 2008-07-17

**Authors:** Mark Schaefer, Mavi Schellenberg, Uta Merle, Karl Heinz Weiss, Wolfgang Stremmel

**Affiliations:** 1 Department of Gastroenterology and Infections Diseases, University of Heidelberg Medical School, INF 410, D-69120 Heidelberg, Germany

## Abstract

**Background:**

In Wilson disease, copper is not sufficiently excreted into bile due to the absence or malfunction of the Wilson protein copper ATPase in the excretory pathway of hepatocytes. Copper is found in sweat. It is unknown if the Wilson protein plays a role in copper excretion into sweat. It is the aim of this study to investigate Wilson protein expression in sweat glands and analysing its effects on copper excretion into sweat in controls and patients with Wilson disease.

**Methods:**

Immunofluorescent analysis of the Wilson protein in skin samples from normal rat, LEC rat and human skin biopsies were performed. Pilocarpin-induced sweat gland stimulation by iontophoretic transfer adapted from the methods used for cystic fibrosis sweat test was used for sweat induction. Sweat volume, sweat copper concentration, serum ceruloplasmin and serum copper were analysed in 28 Wilson patients and 21 controls.

**Results:**

The Wilson protein is expressed in human and rat sweat gland epithelia. Copper concentration in sweat is not significantly different between controls and Wilson patients. Wilson patients produce significantly smaller volumes of sweat compared to controls. Sweat production is partially reversible in Wilson patients under medical treatment for Wilson disease or after liver transplantation

**Conclusion:**

Wilson patients show a reduced sweat production with unaltered sweat copper concentration. The Wilson protein might play an important role in physiological sweat production.

## Background

Wilson disease is an autosomal recessive disease of copper metabolism [1-3]. Mutations in the Wilson gene Atp7b result in an absence or malfunction of the Wilson protein [4]. The Wilson protein is a transmembranous copper ATPase located in the trans-Golgi-network of human cells providing copper to cuproenzyms such as ceruloplasmin. The Wilson protein can move within the cell from the trans-Golgi-network to a late endosomal compartment when intracellular copper concentrations are elevated. In this cellular localization it has an important function in copper excretion out of the cell as copper is excreted via this late endosomal compartment out of the cell. The Wilson protein is mainly expressed in hepatocytes. Biliary excretion of excessive copper is one of the central functions of the Wilson protein [5]. However, Wilson protein is also expressed in other tissues such as kidney, brain, intestine and heart [6]. Patients with Wilson disease usually develop symptoms between the age of 5–70 years. Patients often have hepatic changes due to the copper accumulation in the liver, e.g. liver cirrhosis, fatty liver disease, elevated liver enzymes, acute liver failure. Neurological or psychiatric symptoms can also be seen in Wilson patients (e.g. tremor, gait disturbance, blurred speech, increased mucle tone, depression) [7]. Hyperpigmentations at the lowers legs have been associated as rare dermatological manifestation of Wilson disease [8]. Treatment of Wilson disease with the copper chelating agent D-penicillamine can result in therapy induced changes like loss of elastic fibres, pseudoxanthoma elasticum with elastosis perforans serpiginosa [9], cutis laxa [10] and others summarized as the D-penicillamin-induced degenerative dermatosis [11]. The alternative copper chelator trientine results in a smaller number of skin changes. Copper can be detected in human sweat [12-16]. Copper concentrations in sweat show interindividual variations and to a lesser degree intraindividual variations. Copper concentrations vary depending on the collection area [13]. During exercise, copper sweat excretion is stable [14]. Females show slightly lower copper concentration in sweat than men. However, in situations resulting in higher serum levels of the cuproenzyme ceruloplasmin such as pregnancy or under medication resulting in anovulation for anticonception purposes, women have higher sweat copper levels [15]. In the past, sweat has been collected by applying platic bags or filter papers to certain skin areas and exposing test persons to exercise or humidity and heat (sauna) or by pilocarpin induced ionotopheresis followed by sweat collection with filter papers [16]. The mechanisms of copper excretion into sweat are not characterized so far. Diffusion, passive transport or paracellular transport from surrounding tissues or serum molecules into the sweat, as described previous for other compounds such as ethanol [17] or lactate [18], could be potential mechanisms for copper excretion into sweat. As copper is a highly reactive reagent due to its potential to oxidize or reduce reaction partners, nature has evolved with a number of copper-binding und copper-handling proteins such as Atox1, COMMD1, COX 17, CCS resulting in a directed and safe handling of copper within the cell and the body [6]. Sunderman et al. reported of 2 Wilson patients with reduced sweating during sauna bathing [19]. Therefore this study was aimed to investigate Wilson protein expression in sweat glands and analysing its effects on copper excretion into sweat in controls and patients with Wilson disease. The goals of this study were investigating Wilson protein in sweat glands, developing a scientific method of sweat collection and copper determination in Wilson patients and analysing sweat volumes and copper excretion in Wilson patients and controls as a potential diagnostic tool for Wilson disease.

## Methods

### Patients and controls

The procedures performed within this study both in humans and animals have been approved by the local ethic committees (No. 346/2005) and are in compliance with the Helsinki Declaration. Informed consent was obtained from each patient prior to sweat tests and obtaining blood. As shown in Table 1, the sex distribution and average age of the Wilson patients and controls reflected the age group treated in the institution [7]. In 4 untreated Wilson patients, sweat volume was to low to measure copper concentrations. These patients were not included in the statistical analysis of the sweat copper concentrations. Of the 23 patients under treatment, 15 were treated with D-penicillamin therapy, 4 with trientine, 3 patients with zinc and 1 patient was on a combination therapy of zinc and trientine at the time of the sweat test. Table 2 outlines the detailed data of each patient. Patients 37 and 49 have been measured at 2 time points.

**Table 1:** Patients and controls included in study

	Number	Male/Female	Age ± SD
Wilson patients	28	8/20	32 ± 10
Controls	21	11/10	32 ± 2

**Table 2:** Detailed data of all patients included into the study

No	Group	Age [years]	Sex M = male F = female	Sweat copper concentration [μmol/l]	Sweat volume [μl]	Remarks
1	Control	25	F	2.98	80.0	
2	Control	32	F	1.85	69.0	
3	Control	25	F	2.89	33.0	
4	Control	28	M	1.66	37.4	
5	Control	31	F	2.89	40.0	
6	Control	23	F	2.59	44.0	
7	Control	37	F	1.18	76.0	
8	Control	29	M	2.00	122.0	
9	Control	30	M	5.06	74.0	
10	Control	25	M	1.27	82.0	
11	Control	49	M	1.43	92.0	
12	Control	31	F	2.58	38.0	
13	Control	25	M	2.54	47.0	
14	Control	30	M	1.34	34.0	
15	Control	49	F	5.66	15.0	
16	Control	52	F	2.68	57.0	
17	Control	30	M	2.59	62.0	
18	Control	24	M	1.29	29.0	
19	Control	30	F	1.77	94.0	
20	Control	25	M	1.30	116.0	
21	Control	33	F	2.23	95.0	
22	Wilson patient on medication	54	M	2.03	36.0	D-penicillamin
23	Wilson patient on medication	25	F	1.99	30.9	trientine/zinc
24	Wilson patient on medication	34	F	9.94	25.0	D-penicillamin
25	Wilson patient on medication	19	F	4.93	26.0	D-penicillamin
26	Wilson patient on medication	19	F	2.84	37.0	D-penicillamin
27	Wilson patient on medication	21	M	2.54	41.5	D-penicillamin
28	Wilson patient on medication	25	F	3.97	38.0	D-penicillamin
29	Wilson patient on medication	41	F	0.34	42.2	zinc
30	Wilson patient on medication	36	M	3.02	58.0	D-penicillamin
31	Wilson patient on medication	43	F	0.82	40.2	D-penicillamin
32	Wilson patient on medication	25	M	n.d.	1.0	D-penicillamin
33	Wilson patient on medication	53	F	0.40	70.0	zinc
34	Wilson patient on medication	32	F	1.94	31.0	trientine
35	Wilson patient on medication	38	F	n.d.	1.4	D-penicillamin
36	Wilson patient on medication	24	F	2.75	27.0	D-penicillamin
37	Wilson patient on medication	37	M	2.62	31.6	trientine
38	Wilson patient on medication	31	F	6.74	32.2	trientine
39	Wilson patient on medication	43	F	3.78	53.0	D-penicillamin
40	Wilson patient on medication	46	F	3.18	35.0	D-penicillamin
41	Wilson patient on medication	23	M	1.41	81.0	D-penicillamin
42	Wilson patient on medication	18	F	3.02	38.0	D-penicillamin
44	Wilson patient on medication	24	M	1.49	86.0	trientene
45	Wilson patient on medication	42	M	3.11	36.0	zinc
46	Wilson patient without therapy	33	F	3.00	23.0	no therapy
47	Wilson patient without therapy	18	F	n.d.	6.0	Acute liver failure before liver transplantation
37	Wilson patient without therapy	37	M	n.d.	0.0	initial diagnosis, before therapy
48	Wilson patient without therapy	18	F	n.d.	0.0	Acute liver failure before liver transplantation
49	Wilson patient without therapy	56	F	n.d.	0.3	No therapy, compliance problem
50	Wilson patient without therapy	23	F	n.d.	12.0	Initial diagnosis, before therapy
49	Wilson patient after liver transplantation	20	F	n.d.	46.0	6 months after liver transplantation
51	No Wilson disease Liver transplantation for Budd- Chiari syndrom	20	F	n.d.	57.0	6 months after liver transplantation
52	Liver cirrhosis	44	F	3.07	39.2	
53	Liver cirrhosis	54	M	0.47	79.1	
54	Liver cirrhosis	52	M	1.84	58.0	
55	Liver cirrhosis	44	M	1.81	78.0	
56	Liver cirrhosis	47	M	2.28	53.6	
57	Liver cirrhosis	54	W	2.84	39.7	
58	Liver cirrhosis, PSC	39	M	1.85	46.0	

### Sweat test

A standardized method was developed for studying copper excretion in sweat stimulated by pilocarpin-iontophoreses: Sweat samples were obtained by a standardized protocol at the forearms of patients and controls: The skin was cleaned thoroughly with disinfection solution (isopropanol) and distilled water, respectively. Sweat production was stimulated by a 5 minute iontophoretic transfer (1.5 mA, max.35 V) of 0.5% pilocarpin solution into the skin at an area of about 4 cm^2 ^using a standardized iontophoretic unit used for cystic fibrosis sweat test (Wescor company). After cleaning the test area with destilled water, a modified sweat collector system with an collection area of 3.6 cm^2 ^(Macroduct, Wescor) was applied for 30 minutes. Due to the clinical setting, sweat tests were not standardized for the time of the day when sweat collections were done, the humidity or temperature of the room, the diet of the test persons, even though some of these factors may influence the sweat production. Potential effect on *non*-pilocarpin-dependent sweat production might also influence our results and were not analysed separately. Multiple sources of copper contamination had to be removed from the material commercially available for cystic fibrosis sweat tests including the standard pilocarpin containing agarose-gel discs and the dye making it easier to detect sweat in the collection tube. Every step of the procedure was investigated separatly in order to abolish any kind of contamination during the whole process of sweat simulation, sweat collection and sweat handling/storage. The validity of the results obtained becomes obvious when comparing previous result obtained in studies analysing copper excretion in sweat, either during exercise, during sauna bathing or stimulated by pilocarpin. The copper concentrations obtained in this study are well in the range of the reported data [12-16,19]. The volume of the sweat samples was determined. The copper concentration was measured by atomic absorption. Total copper excretion was calculated (volume × copper concentration).

Out of blood samples obtained during sweat collection, serum copper and ceruloplasmin concentrations were determined by standard laboratory methods.

### Immunofluorescence

For immunofluorescence studies, a polyclonal antibody directed against the copper binding sites 4–6 of the Wilson protein was utilized in frozen sections of rat paws and an human autopsy skin sample embedded in a standard embedding solution (Tissue Tec) as outlined before [5]. Counterstainings were perfomed with Haematoxylin-Eosin and a blue fluorescent DNA-binding dye (Hoechst^®^).

### Statistical analysis

Statistical analyses were performed with SPSS for Windows, release 10.05 (SPSS, Chicago, IL). Comparisons of quantitative variables were performed by the unpaired Mann-Whitney-Test test. Data are means ± SD. A *P *value < 0.05 was considered as statistically significant.

## Results

### Wilson protein in sweat glands

**Figure 1:**
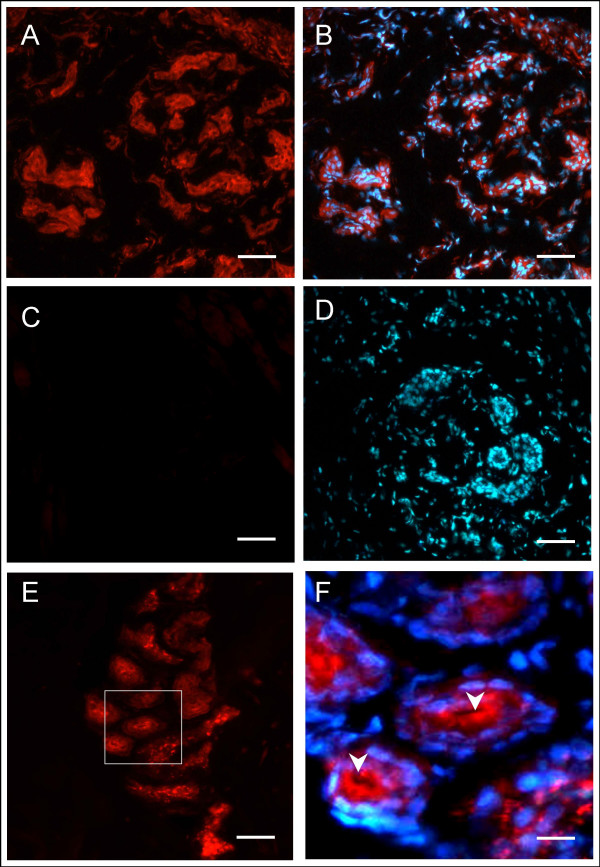
**Wilson protein detection in sweat glands by immunoflourescence**. Frozen sections from normal rat paws (A+B) and LEC rat paws (C+D) and human skin (E-F) were incubated with the following antibodies: A-F: Anti-Wilson Protein Antibody followed by a red fluorescent secondary antibody. B+D+F: DNA couterstain with Hoechst DNA dye. F is the marked detail out of E. Arrows indicate the lumen of the sweat gland. The Wilson protein is abundantly expressed in the secretory and ductular epithelial cells of the sweat glands both in human and normal rat, but not in the LEC rat, which has no expression of the Wilson protein. Magnification: A-D: 100×, E:40×, F: 600×. Bar: A-D 12 μm; E 30 μm; F 2 μm.

Immunofluorescent studies in frozen sections of human and rat skin utilizing a Wilson protein antibody demonstrated abundant expression of the Wilson protein in the secretory and ductular epithelial cells of the sweat glands both in human and rat (Figure 1). There was no signal obtained in control experiments when the Wilson protein antibody as the primary antibody was preabsorbed with the GST-Wilson protein-fusion protein used to raise the antibody or when the Wilson protein antibody as the primary antibody was not added (data not shown). When analysing skin samples from the LEC rat, which does not express the Wilson protein, as negative control, no signal could be obtained in the sweat glands (Figure 1C/D). There was no obvious change of morphology of sweat glands in LEC rat.

To investigate the role of Wilson protein in sweat gland cells, sweat copper concentration and sweat volume was analysed in controls and patients diagnosed with Wilson disease.

### Sweat copper concentration

**Figure 2:**
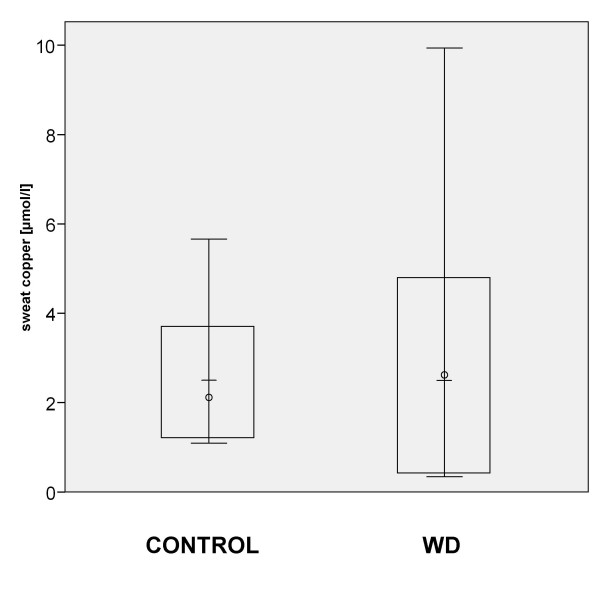
**Sweat copper concentration**. Box blot graphic: Sweat copper concentrations are unaltered in Wilson patients compared to controls. Circle: median. Bar: average. Box : 2× standard deviation.

Copper concentration in sweat was unaltered between the group of Wilson patients compared to controls (Figure 2). Statistical subgroup analysis of untreated Wilson patients was not possible, as only 1 untreated Wilson patients (Table 2 Nr. 45) produced enough sweat volume to determine the copper concentration. The copper concentration determined in this single patient (3,0 μmol/l) was within the box blot range for treated Wilson patients.

### Reduced sweat volume

**Figure 3:**
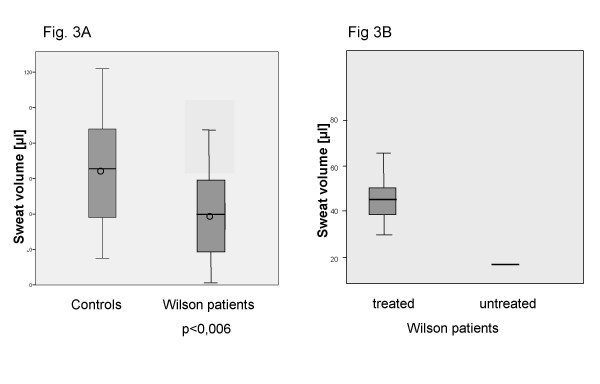
**Sweat volume**. 3A: Sweat volume of controls/Wilson patients Wilson patients produce significantly less sweat during the collection period compared to controls. Wilson patients without detectable sweat volume were not included into the analysed groups. Circle: median. Bar: average. Box : 2× standard deviation. 3B: Sweat volumes of treated Wilson patients and untreated Only 4 of the 6 Wilson patients without treatment produced detectable volumes of sweat. The average sweat production of the 6 patients is given as a bar. Untreated Wilson patients show a very low sweat production. Bar: average. Box : 2× standard deviation.

Wilson patients showed a significant reduction in the sweat volume produced during the 30 min collection time (Figure 3A). Wilson patients without therapy produced even less sweat volume (Figure 3B).

### Total copper excretion

**Figure 4:**
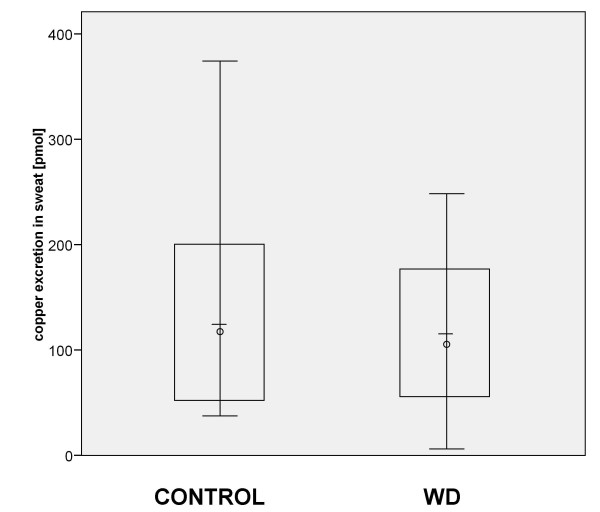
**Total copper excretion**. The total amount of copper excreted into sweat did not differ significantly between the controls and all Wilson patients producing more than 20 μl sweat. Circle: median. Bar: average. Box : 2× standard deviation.

Multiplication of the sweat volume produced during the 30 min collection time with the copper concentration measured in the sweat sample gives the total copper excretion. There was no significant difference of the total excretion of copper in sweat between controls and Wilson patients who produced more than 20 μl of sweat, so total copper excretion could be calculated (Figure 4). There were 5 Wilson patients producing less than 20 μl of sweat (Table 2), so total copper excretion could not be calculated for these patients, as copper concentration could not be determined exactly in these small volumes. 2 Wilson patients did not produce any sweat during the collection time (Table 2). If Wilson patients producing no sweat would be added to the group of Wilson patients for which copper concentration in sweat could be determined, overall total copper excretion is less for Wilson Patients than for controls. This is even more true for the subgroup of untreated Wilson patients.

### Partial reversibility

Obviously, treatment of the disease resulted in an increase in sweat production (Figure 3B). This was not specific to any type of treatment as no correlation between the type of treatment (D-penicillamine/trientine/zinc) and the amount of sweat produced by Wilson patients could be found. During this study, one newly diagnosed patient could be measured before and after 6 months trientine treatment demonstrating the partial reversibility of reduced sweat production in Wilson patients. (Table 3). However, Wilson patients do not reach the levels of sweat excretion of controls (Figure 3). To get further insight into the reversibility of the reduced sweat volume in Wilson patients one additional Wilson patient with fulminant liver failure was analysed before liver transplantation and several weeks thereafter. Whereas no sweat could be obtained before transplantation there was a return of sweat production after transplantation (Table 3). A patient transplanted for Budd-Chiari syndrome without signs of Wilson disease was analysed as a control after liver transplantation.

**Table 3:** Reversibility of impaired sweat excretion.

**Sweat volumes before and after medical treatment**	
Wilson patient (No.37) initial diagnosis before therapy	0 μl
Wilson patient (No.37) after 6 months trientine therapy	31.6 μl
	
**Sweat volumes before and after liver transplantation**	

Wilson patient (No. 49) before liver transplantation	0.3 μl
Wilson patient (No. 49) 6 months after liver transplantation	46.0 μl
Control patient (No. 51) 6 months after liver transplantation	57.0 μl

No linear correlation could be found between the serum copper, serum ceruloplasmin and serum non-ceruloplasmin bound copper and the sweat copper concentration, the sweat volume and the total copper excretion in sweat in all test persons (data not shown). Correlations were tested in pairs (e.g. serum copper- sweat copper concentration in all patients and control). Multifactorial analyses were not performed. Subgroup analyses were not performed.

## Discussion

### Wilson protein expression

The Wilson protein is found in the epithelial cells of the sweat gland. Even though the Wilson protein is mainly expressed in the liver, where it has the essential role of removing excess copper out of the body via the bile and thereby keeping whole body copper balance, the Wilson protein is also expressed in many other tissues, e.g. brain, heart, lung, intestine, placenta and mammary gland [6,20,21]. Under physiological conditions, the function of the extrahepatic Wilson protein seems mainly supply of copper for cellular production of cuproenzyms rather than copper excretion out of the cell [6]. This may also be the physiological role of the Wilson protein within the sweat gland. The Wilson protein might also be responsible for copper supply to cuproenzymes critical for neurotransmitter processing, such as the dopamin β-hydroxylase and tyrosinase [22], as the sweat gland is stimulated by a complex innervation by both parasympathetic and sympathetic nerve fibres with a complex interaction with inhibitory and agonistic signals between the systems and the effector cell [23]. It is an emerging concept for tissues with polarized cells like placenta or intestinum, that both, the Wilson protein and the Menkes protein as copper-ATPases are coexpressed and regulated e.g. by hormonal or metabolic factors [6,24]. Additional investigations might also give insight into a potential interplay between the Wilson protein and its "sister"-protein, the Menkes protein, in the copper metabolism of sweat gland epithelial cells.

### Sweat copper concentration

Sweat copper concentrations are unaltered comparing Wilson patients and controls. This suggests, that the Wilson protein has no essential role in copper excretion into sweat. The mechanisms of copper transport into sweat are unknown so far. Given the increased non-ceruloplasmin bound copper concentrations in serum and tissues as well as the generally elevated copper concentrations in nearly all tissues in Wilson patients, elevated copper concentrations would to be expected in sweat of Wilson patients if copper excretion into sweat would follow simple diffusion, passive transport or paracellular transport from tissue or serum compounds into the sweat, as described previous for other compounds such as ethanol [17] or lactate [18].

Obviously, this study is limited as it could only measure copper concentration and the volume of the final sweat as it gets to the surface of the skin. It is possible that concentrations of metals and electrolyes vary between the primary sweat fluid and the sweat secreted onto the skin due to reabsorption and active secretion in the sweat ducts. To analyse this in more details, studies utilizing isolated sweat glands would be a potential way for investigations.

### Reduced sweat volume

The reduced sweat volume, which is especially extreme in untreated patients, could be explained by cellular dysfunction: Due to the reduced physiological function of the Wilson protein or due to toxic, damaging effects caused by the mutated protein (e.g. entrapment in the ER or formation of aggregates) as described previously for the Wilson protein [25] and also described in other diseases with impaired sweat productions in sweat gland cells, e.g. α-galactosidase deficiency, Fabrys disease [26], sweat production is impaired. Wilson protein malfunction might also effect the function of chloride transporters which might play a role in sweat production. Colocalization and coimmunoprecipitation of the Wilson protein and the Chlorid transporter ClC-4 as well as effects of the expression of ClC-4 on Wilson protein function have been demonstrated before [27]. Inhibitory effects of elevated cellular copper concentrations due to the missing cellular excretory function of the Wilson protein could also explain the reduction in sweat volumes in affected patients. However, as cellular copper concentrations could not be analysed in this study, this remains speculative. Potential influences of additional factors e.g. hypoproteinemia, age, race and skin conditions have not been analysed in this pilot study but might be relevant [28].

On the other hand, the data shows a clear increase of sweat volume when the Wilson disease patients are treated either by medication (D-penicillamine/trientine/zinc) or after liver transplantation (Figure 3B, Table 3). Under either treatment, the malfunction or absence of the Wilson protein within the epithelial sweat gland cell remains unchanged. However, liver transplantation results in an excretion of excessive and accumulated copper via the biliary fluid normalizing whole body copper concentrations over time. Medical treatment with the chelating agents D-penicillamine and trientine or treatment with zinc, which blocks copper absorption and induces copper-binding metallothionin within the body, all result in a netto efflux of copper out of the body and lowering of non-ceruloplasmin bound copper concentrations all over the body. This reduction of toxic copper and overall and cellular copper load allows damaged cells to regenerate and function normally. The same may be true for the sweat gland cells. Especially hepatocytes and neuronal cells, which are mainly affected during the course of Wilson disease benefit from Wilson medications or transplantation. One might even speculate, that impairment and partial regeneration of the autonomous nervous system innervating the sweat gland cells by the complex sympathetic postganglionar cholinergic innervation of the sweat gland, explains the reduced and partially reversible sweat production in Wilson patients. Malfunction of the autonomous nerve system and their partial or complete normalization have been described in Wilson patients for regulation of cardiovascular functions, saliva production and sympathetic skin response [3,29,30] before.

Even though the sweat test is a non-invasive test, it does not add any clinical value to the diagnosis of Wilson disease as the established serum markers ceruloplasmin and copper were more accurate in differentiating controls from Wilson patients.

## Conclusion

The Wilson protein is expressed in the sweat gland epithelial cells. In Wilson disease, there is an impairment of sweat production. Impairment of sweat production can be improved by standard medical therapy for Wilson disease or by liver transplantation. Wilson patients do not compensate for reduced biliary copper excretion by an increased sweat copper excretion. The impairment of sweat production might be of relevance for thermoregulation for Wilson patients.

## Competing interests

The authors declare that they have no competing interests.

## Authors' contributions

MSchaefer has designed and coodinated of the study, prepared the manuscript and was active in aquisition of patients, MSchellenberg carried out the sweat tests, UM and KHW did the statistical analysis, UM, KHW and WS participated in the design and coordination of the study and helped to prepare the manuscript. All authors read and approved the final manuscript.

## Pre-publication history

The pre-publication history for this paper can be accessed here:


